# Factors associated with cervical cancer screening among young unmarried Japanese women: results from an internet-based survey

**DOI:** 10.1186/s12905-018-0623-z

**Published:** 2018-07-31

**Authors:** Noriyo Kaneko

**Affiliations:** Department of Global and Community Health, School of Nursing, Kawasumi 1, Aza, Mizuho-cho, Mizuho-ku, Nagoya, Aichi Japan

**Keywords:** Cervical cancer, Cervical cancer screening, Young women, Internet survey, Sexual behavior, HPV

## Abstract

**Background:**

The prevalence of cervical cancer among women aged 20–30 years has been increasing. A better understanding of the factors correlated with cervical cancer screening is vital to better identify suitable candidates and develop effective interventions. However, few studies have examined factors correlated with cervical cancer screening using a quantitative research design. Thus, this study aimed to assess the prevalence of and factors correlated with cervical cancer screening among unmarried and sexually active Japanese women aged 20–29 years.

**Methods:**

Seven hundred Japanese women who responded to an internet-based cross-sectional survey conducted by a marketing research company in 2015 were enrolled. Associations between lifetime cervical cancer screening and demographic profile, sexual behavior, and psychosocial factors were assessed via univariate analysis. Variables indicating significance (*P* < 0.05) were used in the univariate analysis to determine adjusted odds ratios (AOR).

**Results:**

Overall, 383 (54.7%) respondents underwent cervical cancer screening during their lifetime. Multiple regression analysis indicated that age, employment status, income, lifetime number of sex partners, human papillomavirus (HPV) vaccination, receipt of a free coupon for cervical cancer screening from the local government, perceived susceptibility and logistical barriers (cost/time), and confidence of receiving Pap testing from a male physician were significantly correlated with lifetime cervical cancer screening. Individuals aged 28–29 years (AOR = 1.86) and those with full-time employment (AOR = 3.30), income ≥ ¥ 4,000,000($35,000) (AOR = 1.60), > 5 lifetime sex partners (AOR = 1.97), HPV vaccination (AOR = 4.88), coupon from the local government (AOR = 3.14), higher perceived level of cervical cancer susceptibility (middle, AOR = 1.77; high, AOR = 3.23), lower perceived logistical barriers (middle, AOR = 0.55; high, AOR = 0.31), and higher confidence of receiving pap testing from a male physician (AOR = 2.66) were more likely to undergo cervical cancer screening.

**Conclusions:**

Women who were younger and unemployed and those with lower perceived cervical cancer susceptibility, higher perceived logistical barriers, and lower confidence of receiving Pap testing from a male physician were less likely to undergo lifetime cervical cancer screening. Thus, to increase the cervical cancer screening rate among young women, it may be effective to target younger unemployed women, provide interventions to increase perceived susceptibility, and recommend tests while considering psychosocial barriers.

**Electronic supplementary material:**

The online version of this article (10.1186/s12905-018-0623-z) contains supplementary material, which is available to authorized users.

## Background

Cervical cancer is the most common type of cancer among women aged 20–29 years in Japan. About 9390 new cervical cancer cases are diagnosed annually in Japan (estimations for 2012). Cervical cancer is the second most common female cancer in women aged 15 to 44 years in Japan, with an age-standardized incidence rate per 100,000 of 10.9, which is higher than the East Asia average. Approximately, 3645 cervical cancer deaths occur annually in Japan (estimations for 2012), and the age-standardized mortality rate is 2.8 [1]. Moreover, the prevalence of cervical cancer among women aged 20–30 years has been constantly increasing [2–4].

Several studies have confirmed that regular cervical cancer screening leads to early detection and a decrease in the mortality rate of individuals with cervical cancer [5]. In Japan, cervical cancer screening is conducted at obstetrics and gynecology departments in hospitals or clinics, and not at health centers. Furthermore, only physicians can conduct Pap smear tests. These guidelines are developed by the Japanese Advisory Committee on Cancer Screening and are implemented by local governments. In the national screening guidelines, cervical cancer screening using conventional and liquid-based cytology is performed for population-based and opportunistic screening every 2 years in women aged 20 years and older. In Japan, pregnant women undergo cervical cancer screening during gynecologic checkups [6]. For women who attend the obstetrics and gynecology clinic to receive oral contraceptive pills or because of sexually transmitted infections, cervical cancer screening is not mandatory, but it is positioned as a test to be conducted as necessary.

In Western countries, including the United States and the United Kingdom, the rate of undergoing cervical cancer screening in a lifetime is > 80%. However, in Japan, the lifetime rate is only 42.1% [7]. In particular, the lifetime screening rate for women aged 20–29 years is 22.2%, which is a cause of concern [8]. To address this, a free-coupon program for cervical cancer screening was introduced in Japan in 2009 as a national policy. Under this program, a coupon or voucher for a free cervical cancer screening was sent by mail to women aged 20, 25, 30, 35, and 40 years [9]. However, the screening rate for young women is still critically low.

In developed countries, the human papilloma virus (HPV) vaccine is widely administered to young women to prevent the development of cervical cancer; it has been effective in reducing HPV infection and associated disease among the young population nationwide and in lowering the long-term incidence of HPV-related cancers [10, 11]. However, in Japan, due to unconfirmed severe side effects, the national promotion of such vaccines has been suspended [12]. Hence, screening as a preventive measure for cervical cancer among the young population has become extremely important.

A better understanding of factors correlated with the behavior of individuals toward cervical cancer screening to prevent the condition is essential to identify suitable candidates and develop effective interventions.

Recent studies have assessed the perception of individuals regarding cervical cancer screening as well as the effectiveness of the coupon provided by the government that aims to increase the screening rate among women aged 20–30 years [13]. A qualitative research study on Japanese university students showed that low perceived susceptibility to the disease, lack of knowledge and motivation, and reluctance to visit a gynecologist are possible barriers of cervical cancer screening [14]. Moreover, in another quantitative research study, inconvenience, inadequate time, reluctance to undergo the screening procedure, and high cost were found to be associated with not undergoing cervical cancer screening among women aged 20–59 years [15]. Additionally, a low education level, lack of knowledge regarding the signs and symptoms of cervical cancer, low perceived behavioral control, and negative feelings, such as stigma and shame, have been considered as barriers among individuals to undergo a Pap test in other Asian countries [16–19].

However, factors that predict the behavior of young unmarried women toward cervical cancer screening in Japan remain unclear. In the past decade, no quantitative research study has examined factors correlated with behavior toward cervical cancer screening in unmarried women in Japan who have never been pregnant. In fact, women aged 20–29 years, particularly unmarried women with sexual experience but who have never been pregnant, represent the target group for the promotion of cervical cancer screening.

In the present study, the prevalence and factors correlated with cervical cancer screening among unmarried Japanese women aged 20–29 years were assessed.

## Methods

### IRB approval

The study protocol was approved by the Research Ethics Committee of the School of Nursing, Nagoya City University, Japan.

### Sample and data collection

The web-based survey was cross-sectional in design. The target population was unmarried Japanese female participants aged 20–29 years who had no history of pregnancy and had the experience of sexual intercourse. The sample was randomly selected from individuals who were willing to participate in the survey conducted by a research company. No personal identifying information was collected. The survey site was hosted, and all data were stored on a secure server.

The survey was administered via a website, created solely for this research project. Visitors interested in the study were asked to click on the banner, upon which they were transferred to an information page describing the survey including information on the length of the survey, purpose of the study, the investigator and her affiliation, how data would be stored, and how the results would be published. Those who understood the research details were asked to click on the link, “I understand all conditions and agree to participate in the study”, which led to the questionnaire page. The survey was voluntary. Respondents could leave the website without completing the survey.

A total of 700 Japanese women aged 20–29 years who responded to the internet-based, cross-sectional survey that was conducted by a reliable research company, which is a major portal site company in Japan, were included in the study. The survey was closed to individuals willing to participate in surveys provided by the research company associated with the major portal site company in Japan. This research company offers full-scale marketing services, covering approximately 2,280,000 individuals who are willing to participate in surveys across Japan; demographic data, including age, sex, and residential area, were obtained. Thus, the company can select specific sociodemographic groups from the registered population based on the inclusion criteria of each survey. To obtain data that match the inclusion criteria of this study, participants underwent two stages of recruitment. Initially, potential respondents (*N* = 81,553) who were female, aged 20–29 years old, and unmarried were randomly and blindly selected and invited to participate in the first stage of the survey via e-mail. The e-mail with the invitation message contained a URL that directed the responders to a secure page on the website, which required access using a unique log-in ID and password. Invitation mails were sent only to registered accounts, and to enter the survey site, respondents had to enter his/her own ID and password. With ID and password authentication, duplicate access was blocked.

Participants were asked about their marital status, educational background, occupation, and experience of sexual intercourse, pregnancy, and giving birth. At the first stage of the recruitment process, 81,553 e-mails were sent, and 10,000 accounts answered the first stage questionnaire for screening. However, the first stage survey was automatically closed at the point when 10,000 respondents were registered. Of the 10,000 participants, 6070 met the inclusion criteria. The second e-mail invited the participants to join the second stage of the survey, and this email was sent to 6070 participants. After 700 participants voluntarily completed the questionnaire, exclusive access to the website was restricted. Only completed questionnaires were used for this study. Completeness was checked for each question. If there was any incomplete item, survey system automatically requested the participant to complete the item and proceed to the next page. As an incentive, the internet research company provided up to 200 reward points to the participants, which is equivalent to 200 yen ($1.75 in United States Dollars as of November 6, 2017). The survey was conducted on March 27, 2015.

### Item development and testing

The survey items were developed for this study. Qualitative findings from a previous study were used for developing the survey items for this study. The questionnaire was reviewed by four Japanese volunteers. The online survey was pretested, and correct functionality was checked before initiating the formal survey. The final number of questionnaire items was 18, and the total number of web pages was 9. For details regarding the final survey items, please refer to Additional file 1.

### Measurements

#### Prevalence of lifetime cervical cancer screening and sociodemographic characteristics

Participants were asked if they had undergone cervical cancer screening during their lifetime (yes/no). Possible sociodemographic characteristics potentially correlated with the cervical cancer screening experience included age (20–23, 24–27, and 28–29 years), education level (below high school, 3 years in college/junior college, or university level), possession of medical- and health-related licenses such as medical, nursing, pharmaceutical science areas (yes/no), employment status (unemployed or student and with part-time or full-time job), income (< 2,000,000, < 4,000,000, or ≥ 4,000,000 yen, which is equivalent to < 18,110, < 36,820, or ≥ 36,820 in US dollars [USD]), routine hospital visit (yes/no), and history of lifetime smoking (yes/no).

#### Sexual practice and social and psychological factors

With regard to sexual practice, participants were asked about the number of sex partners thus far (1, 2–4, or > 5), history of contracting sexually transmitted infections (STIs) (yes/no), knowledge on cervical cancer (yes/no), HPV vaccination (yes/no), and receipt of coupon for cervical cancer screening from the local government (yes/no).

The perceived susceptibility to cervical cancer was measured on a 2-item scale (1: no problems as my friends do not have cervical cancer; 2: the possibility of developing cervical cancer is low at my age), whereas the perceived logistical barriers of cervical cancer screening were measured on a 3-item scale (1: no time for cervical cancer screening; 2: it is inconvenient to undergo cervical cancer screening; and 3: it is expensive to undergo cervical cancer screening). A 5-point Likert-type response format was used to rate the items (1: strongly disagree, 2: somewhat disagree, 3: neither agree nor disagree, 4: somewhat agree, and 5: strongly agree) for perceived susceptibility and logistical barriers. The Cronbach’s alpha values were 0.72 and 0.67 for the perceived susceptibility and barriers, respectively. For scores of perceived susceptibility and logistical barriers, first, the mean score for each was calculated, and based on the distribution of the data, three division points were defined and categorized into 3 levels (low, middle, or high) for each scale.

The confidence level of individuals who believed that a male physician would conduct the cervical cancer screening was classified as high, middle, or low.

### Statistical analysis

The associations between demographic profiles, sexual practice, and social and psychological factors and lifetime cervical cancer screening were assessed via a univariate analysis, using the Pearson’s chi-squared test or Mann–Whitney U test. Factors with a *P* value < 0.05, which was considered statistically significant in the univariate analysis, were used in the multivariate analysis to determine the adjusted odds ratios (AOR). The 95% confidence intervals (95% CIs) were also calculated. A two-sided significance level of 0.05 was used. All statistical analyses were conducted using the SPSS version 19.0 for Windows.

## Results

### Demographic profile and lifetime cervical cancer screening experience

Table 1 presents the demographic profile and lifetime cervical cancer screening experience of the study participants.

**Table 1:** Demographic profile of the study participants (*N* = 700)

	Lifetime experience of pap smear testing				*P* value
	Yes (*n* = 383)		No (*n* = 317)		
	*n*	(%)	*n*	(%)	
Age (years)					
20–23	53	13.8%	76	24.0%	< 0.001
24–27	181	47.3%	148	46.7%	
28–29	149	38.9%	93	29.3%	
Education					
Below high school level	73	19.1%	83	26.2%	0.07
3 years in college/junior college	76	19.8%	60	18.9%	
University level	234	61.1%	174	54.9%	
Possession of medical- and health-related licenses					
Yes	66	17.2%	39	12.3%	0.07
No	317	82.8%	278	87.7%	
Employment status					
Unemployed/student	12	3.2%	22	7.0%	< 0.001
Part-time job	66	17.4%	104	33.2%	
Full-time job	302	79.5%	187	59.7%	
Income					
< 2,000,000 yen	129	33.7%	159	50.2%	< 0.001
< 4,000,000 yen	184	48.0%	135	42.6%	
≥4,000,000 yen	70	18.3%	23	7.3%	
Routine hospital visit					
Yes	106	27.7%	69	21.8%	0.07
No	277	72.3%	248	78.2%	
Smoking history (lifetime)					
No	82	21.4%	59	18.6%	0.36
Yes	301	78.6%	258	81.4%	

A total of 700 participantswere assessed. Among the participants, 383 had undergone Pap smear testing during their lifetime. Participants’ ages ranged from 20 to 29 years (mean age: 26.0 years), and most were 24–27 years. The associations between lifetime cervical cancer screening and demographic factors are presented in Table 1. Among all demographic variables, a significant difference was observed between lifetime cervical cancer screening experience and age, employment status, and income.

### Sexual behavior, psychosocial factors, and cervical cancer screening experience (Table 2)

Cervical cancer screening experience was found to be associated with the lifetime number of sex partners, history of contracting STI, HPV vaccination, receipt of a free coupon for cervical cancer screening from the government, perceived susceptibility and barriers, and confidence of receiving Pap smear testing from a male physician.

**Table 2:** Sexual practice and social and psychological factors affecting the study participants (*n* = 700)

	Lifetime experience of pap smear testing				*P* value
	Yes (*n* = 383)		No (*n* = 317)		
	*n*	(%)	*N*	(%)	
Lifetime number of sex partners					
1	73	19.1%	107	34%	< 0.001
2–4	158	41.3%	123	39%	
> 5	152	39.7%	87	27%	
Concerns regarding STI					
Yes	163	42.6%	99	31%	< 0.01
No	220	57.3%	218	69%	
Knowledge on cervical cancer obtained from school					
Yes	121	31.6%	84	27%	0.140
No	262	68.4%	233	74%	
HPV vaccination					
Yes	43	11.2%	12	4%	< 0.001
No/do not know	340	88.8%	305	96%	
Receipt of a coupon for cervical cancer screening from the local government					
Yes	308	80.4%	172	54.3	< 0.001
No	75	19.6%	145	45.7	
Perceived susceptibility to cervical cancer					
High	214	55.9%	96	30.3%	< 0.001
Middle	124	32.4%	139	43.8%	
Low	45	11.7%	82	25.9%	
Perceived logistical barriers to cervical cancer screening					
High	106	27.7%	169	53.3%	< 0.001
Middle	139	36.3%	103	32.5%	
Low	138	36.0%	45	14.2%	
Confidence of undergoing cervical cancer screening conducted by a male physician					
Low	73	19.1%	109	34.4%	< 0.001
Middle	103	26.9%	116	36.6%	
High	207	54.0%	92	29.0%	

*STI* sexually transmitted infections, *HPV* human papillomavirus

### Factors associated with the lifetime experience of cervical cancer screening (Table 3)

Respondents aged 28–29 years were more likely to have undergone cervical cancer screening (AOR: 1.86; 95% CI: 1.08–3.21) than those aged 20–23 years. Moreover, respondents with an income of 4,000,000 yen were more likely to have undergone the screening (AOR: 3.32; 95% CI: 1.55–7.29) compared to those with an income of < 2,000,000 yen. Respondents who were working full time were more likely to have undergone the screening (AOR: 3.30; 95% CI: 1.46–7.45) than were students or those who were unemployed. Respondents with > 5 sex partners during their lifetime were more likely to have undergone the screening (AOR: 1.97; 95% CI: 1.22–3.20) than those with only one sex partner. HPV vaccination (AOR: 4.88; 95% CI: 2.19–10.89) and receipt of a free coupon for Pap smear testing from the government (AOR: 3.14; 95% CI: 2.12–4.64) were positively associated with the screening experience. Respondents who had a high perceived susceptibility to cervical cancer were more likely to have undergone the screening (AOR: 4.06; 95% CI: 2.63–6.28) than were those with a low perceived susceptibility. Moreover, this association became stronger as the level of perceived susceptibility increased. Respondents with high perceived logistical barriers of Pap smear testing were less likely to have undergone the screening (AOR: 0.31; 95% CI: 0.19–0.50) than those with low perceived barriers. This association became stronger as the level of perceived barriers increased. Respondents with a high level of confidence for Pap smear testing conducted by a male physician were also more likely to have undergone the screening (AOR: 2.66; 95% CI: 1.70–4.19) than those with a low confidence level.

**Table 3:** Multivariate logistic regression analysis of potential associated factors and lifetime Pap smear testing experience (N = 700)

	Have undergone pap smear testing	OR	(95% CI)	P value	AOR	(95% CI)	*P* value
Total	383						
Age (years)							
20–23	53	1	(ref)		1	(ref)	
24–27	181	1.75	1.16–2.65	< 0.01	1.44	0.85–2.44	0.17
28–29	149	2.3	1.49–3.55	< 0.001	1.86	1.08–3.21	< 0.05
Employment status							
Unemployed/student	12	1	(ref)		1	(ref)	
Part-time job	66	1.1	0.54–2.23	0.859	1.91	0.82–4.43	0.740
Full-time job	302	2.8	1.25–5.42	< 0.005	3.30	1.46–7.45	< 0.01
Income							
< 2,000,000 yen	129	1	(ref)		1	(ref)	
< 4,000,000 yen	184	1.68	1.22–2.32	< 0.005	0.92	0.58–1.47	0.13
≥4,000,000 yen	70	3.75	2.22–6.34	< 0.001	1.60	0.83–3.09	< 0.001
Lifetime number of sex partners							
1	73	1	(ref)		1	(ref)	
2–4	158	1.88	1.29–2.75	< 0.005	1.39	0.89–2.17	0.15
≥5	152	2.56	1.72–3.81	< 0.001	1.97	1.22–3.20	< 0.01
Concerns regarding STI							
Yes	220	2.08	1.39–3.13	< 0.005	1.34	0.92–1.95	
No	163	1	(ref)		1	(ref)	0.13
History of HPV vaccination							
Yes	340	3.22	1.66–6.21	< 0.001	4.88	2.19–10.89	
No/do not know	43	1	(ref)		1	(ref)	< 0.001
Receipt of a coupon for cervical cancer screening from the local government							
Yes	308	5.03	3.23–7.81	< 0.001	3.14	2.12–4.64	
No	75	1	(ref)		1	(ref)	< 0.001
Perceived susceptibility to cervical cancer							
Low	45	1	(ref)		1	(ref)	
Middle	124	1.63	1.05–2.52	< 0.05	1.77	1.04–3.01	< 0.05
High	214	4.06	2.63–6.28	< 0.001	3.23	1.89–5.51	< 0.001
Perceived logistical barriers to cervical cancer screening							
Low	138	1	(ref)		1	(ref)	
Middle	139	0.46	0.57–0.33	< 0.001	0.55	0.34–0.90	< 0.05
High	106	0.22	0.14–0.31	< 0.001	0.31	0.19–0.50	< 0.001
Confidence of undergoing cervical cancer screening from a male physician							
Low	109	1	(ref)		1	(ref)	
Middle	116	1.33	0.89–1.97	0.19	1.34	0.85–2.13	0.21
High	92	3.36	2.29–4.94	< 0.001	2.66	1.70–4.19	< 0.001

*OR* adjusted odds ratio, *STI* sexually transmitted infections, *HPV* human papillomavirus

## Discussion

To the best of our knowledge, factors correlated with lifetime cervical cancer screening among sexually active unmarried women aged 20–39 years in Japan were first assessed in this internet-based study. In Japan, the increased prevalence of cervical cancer and remarkably low prevalence of Pap smear testing (approximately 25%) among young women are major concerns of the government. Nevertheless, studies regarding potential factors correlated with the behavior of individuals toward cervical cancer screening are limited. Thus, we conducted an internet-based research study to obtain basic data concerning factors correlated with the experience on lifetime Pap smear testing, and an effective program for young women who are at risk of contracting HPV was then developed.

Sample representativeness is a primary concern concerning web-based surveys. Regarding internet access in Japan, it is reported that 99.2% of women in their 20s have internet access, and there was no gap between rural and urban areas in 2017 [20]. Since the company disclosed information on sociodemographic distribution of all women registered with the company, it was possible to compare the profile between women who were surveyed and women registered with the company: there were more women residing in Tokyo among the study sample compared to those registered with the company. This bias comes from the fact that this survey limits the target to women who are not married, and the unmarried rate of young women is highest in Tokyo. However, there was no significant difference regarding the urban to rural ratio between women surveyed and those registered with the company.

With regard to the cervical cancer screening experience of the respondents, the screening rate was higher in our sample compared to the national value (approximately 32.7% among women aged 20–69 years in 2013). The university graduation level of the sample was 58%. This percentage is higher than national average of 50%, which is the national university graduation rate for the age group of this sample [21]. Access to medical care and preventive service is better for those with university education or higher, and this group in Japan tends to be healthier [22]. Therefore, a higher educational background might lead to a higher cervical cancer screening rate than that in a national sample. To increase the generalizability of the study, the use of nationally representative data is crucial.

In terms of socioeconomic background, age, employment status, and income level were associated with the behavior of individuals toward lifetime cervical cancer screening. Full-time workers and those with an income > 4,000,000 yen were more likely to have undergone cervical cancer screening; this result is consistent with that of previous studies, wherein permanent workers and higher socioeconomic class members were more likely to undergo cancer screening [22, 23]. In particular, full-time workers have more opportunities to undergo health checkups and be provided with health-related information by the company, both of which may result in the higher screening rate among full-time workers.

In terms of sexual experience, the number of sex partners (> 5) during a women’s lifetime was positively associated with behavior toward lifetime cervical cancer screening. This association may derive from the fact that women who have more sex partners are more likely to access obstetric and genecology clinics to access oral contraceptive pills or STI testing. Although cervical cancer screening is not mandatory during attendance for contraception, it is recommended to offer such screening. Future studies need to assess if the access to obstetric and genecology clinics affect Pap smear testing behavior. Although a previous study showed an association between lifetime cervical cancer screening and the number of recent sex partners [24], the association between cervical cancer screening behavior and the number of lifetime sex partners was not clearly elucidated. As the risk of acquiring HPV and cervical abnormalities increases with an increase in the number of sex partners [25], the association between the number of lifetime sex partners and Pap smear testing behavior should be further studied.

The history of HPV vaccination was also positively associated with the behavior of individuals toward Pap smear testing. Guidelines concerning HPV vaccination that were established by the Japanese government strongly recommend cervical cancer screening, even after HPV vaccination. Hence, a patient’s visit for HPV vaccination in the clinical setting can lead to simultaneous Pap smear testing after the recommendation of a physician. However, proactive recommendations for HPV vaccination have been suspended in Japan due to unconfirmed adverse events related to the vaccination. Moreover, the rate of HPV vaccination among young Japanese has also subsequently declined [26]. Such a decrease in the vaccination rate may also lead to a reduction in the rate of cervical cancer screening. Therefore, intervention is needed to disseminate information concerning the importance and necessity of regular cervical cancer screening, using media channels widely used by young people. This is especially true since the cessation of proactive recommendations for HPV vaccination. Moreover, previous studies showed the effectiveness of the use of invitation letters to promote screening [27]. Therefore, the use of invitation letters in addition to the free coupon program that are currently being conducted might be a promising approach.

Respondents with greater perceived susceptibility to cervical cancer were more likely to undergo cervical cancer screening. A previous study showed that the perceived susceptibility of contacting cervical cancer was strongly associated with the cervical cancer screening experience [14]. In the Health Belief Model, perceived susceptibility to a certain disease may be affected by knowledge of that disease [28]. Hence, the dissemination of facts, such as an increase in the incidence of cervical cancer among young women in Japan or that all women who are sexually active can possibly contract cervical cancer, can help increase awareness of the perceived susceptibility to cervical cancer and may result in the increased rate of screening.

Among factors assessed in the present study, the receipt of a free coupon for cervical cancer screening from the local government was significantly associated with lifetime cervical cancer screening experience. A previous study found that the use of government coupons promoted an individual’s behavior toward cervical cancer screening [13]. To continue and enhance this program, promotions based on effective target segmentation may positively affect an individual’s behavior toward screening.

However, the present study also has certain limitations. First, participants who were registered with the research company and willing to participate in surveys were recruited in the study. Selection bias is a major concern in internet-based surveys since it can limit the generalizability of the study. Therefore, selection bias may have limited the generalizability of the results of this survey. However, the random selection of participants, including an equally distributed group aged between 20 and 39 years from a pool of 81,553 individuals, may have helped mitigate bias and ensure greater representativeness in the survey. Moreover, when the participants were asked about personal behaviors, such as sexual behaviors, an internet-based survey enables them to provide more truthful answers compared to a pencil and paper-based questionnaire. Thus, such technique can mitigate the weakness of the study in terms of sample selection. In addition to the limitations related to the internet-based research design, the present study also had other limitations. First, the cross-sectional design did not allow any conclusions regarding causal relationships to be drawn. We speculated that several factors, such as perceived susceptibility; perceived barriers in terms of cost, time, and process; and confidence level of individuals in specific situations, might have affected the individual’s behavior toward Pap smear testing. However, causal relationships and other confounding factors were not identified based on the data that were obtained. Second, all data in the present study were self-reported. With self-reported data, there is a risk of recall bias. Moreover, there is always a social desirability effect when assessing the behaviors and attitudes that are associated with sensitive behaviors.

## Conclusions

In conclusion, the results of the present study suggest that several sociodemographic factors, sexual practices, HPV vaccination history, and psychosocial factors may affect the cervical cancer screening experience, thus offering insights into the predictors of cervical cancer screening behavior among young sexually active unmarried women in Japan. Women who were younger and unemployed and those with a lifetime lower perceived susceptibility to cervical cancer, higher perceived logistical barriers, and lower confidence level regarding Pap smear testing conducted by a male physician were less likely to undergo lifetime cervical cancer screening. Thus, to promote cervical cancer screening, young and unemployed women should be targeted. Moreover, interventions that aim to increase perceived susceptibility must be introduced, and tests that consider the patient’s psychosocial barriers should be conducted.

## Additional file

Additional file 1: Questionnaire. (DOCX 44 kb)

## Abbreviations

AOR: Adjusted odds ratio
HPV: Human papillomavirus
STI: Sexually transmitted infections
