# Supplementary material for: Factors associated with cervical cancer screening among young unmarried Japanese women: results from an internet-based survey
Source: BMC Womens Health. 2018 Jul 31;18:132. doi: 10.1186/s12905-018-0623-z (PMC6069882; doi:10.1186/s12905-018-0623-z)
Supplement: Supplementary file 1 — Questionnaire. (DOCX 44 kb) [file 12905_2018_623_MOESM1_ESM.docx]

**Questionnaire**

**Survey on Cervical Cancer Screening among Women**

The purpose of this questionnaire is to ask about awareness of sexually transmitted diseases and actual cervical cancer screening and to obtain basic data for promoting cervical cancer screening. The survey asks about private things, but since it is conducted anonymously, there is no way that individuals can be identified or personal information leaked to the outside.

Please be assured that all data from you are statistically processed as percentage of the responses, so no personal response will be made public. In addition, we will never use the data for purposes other than for this research purpose. We would be pleased to hear your opinions and ideas.

It takes about 10 minutes to answer the questionnaire. Thank you again for your cooperation and understanding of the purpose of the survey.

First of all, I will ask about yourself.

Q1. Please answer your final academic level.

1. Elementary school
2. Junior high school
3. High school
4. Vocational school
5. Junior college/college of technology
6. University
7. Graduate School
8. Other ( )

Q2. Are you a graduate of or a student at a school where you can obtain health-related qualifications related to medicine, nursing, or pharmaceutical science?

1. Yes
2. No

Q3. Which of the following is closest to your current job status?

1. Full-time (regular employment)
2. Full-time (non-regular employment)
3. Part-time
4. Day-job
5. Business manager
6. Student
7. No job

Others

Q4. Have you ever had sexual intercourse with men in your lifetime?

1. Yes
2. No

Q5. Are you married?

1. Yes
2. No

Q6. Are you pregnant now?

1. Yes
2. No

Q7. Have you ever had experienced giving birth to a child in your lifetime?

1. Yes
2. No

Q8. What is your annual income?

1. Less than 1 million yen
2. 1 million yen or more to less than 2 million yen
3. 2 million yen or more to less than 4 million yen
4. 4 million yen or more to less than 6 million yen
5. 6 million yen or more

Q9. Please tell me about your smoking experience.

1. I am currently smoking
2. I used to smoke, but not now
3. I have never smoked

Q10. Do you currently have some sickness and regularly visit hospitals and clinics?

1. Yes
2. No

Q11. The information below is extremely confidential, so please answer.

Please tell me about how many men with whom you have had sexual experiences so far.

1. 1
2. 2
3. 3
4. 4
5. 5
6. 6
7. 7
8. 8
9. 9
10. Over 10

Q12. Have you ever been worried about sexually transmitted diseases (venereal diseases, diseases caused by sexual intercourse)?

1. Yes
2. No

Q13. Have you learned about cervical cancer (vaccination and prevention) when you were in middle school, high school, or university?

1. Yes
2. No
3. Don’t know

Q14. Have you ever received a coupon for cervical cancer screening sent from a local government?

1. Yes
2. No

Q15. Have you ever had a cervical cancer screening examination?

1. Yes
2. No

Q16. Have you ever received a cervical cancer preventive vaccine (HPV vaccine)?

1. Yes
2. No
3. Don’t know

Q17 .What do you think about the following ideas regarding cervical cancer? Please answer based on five possible responses.

|  | Strongly agree | Somewhat agree | Neither agree nor disagree | Somewhat disagree | Strongly disagree |
| --- | --- | --- | --- | --- | --- |
| 1. There is no problem, as my friends do not have cervical cancer. |  |  |  |  |  |
| 1. The possibility of developing cervical cancer is low at my age. |  |  |  |  |  |
| 1. There is no time for cervical cancer screening. |  |  |  |  |  |
| 1. It is inconvenient to undergo cervical cancer screening. |  |  |  |  |  |
| 1. It is expensive to undergo cervical cancer screening. |  |  |  |  |  |

Q18. How much confidence do you have that cervical cancer screening would be conducted by a male physician?

1. Almost certainly I can undergo it
2. Maybe I can undergo
3. Neither agree nor disagree
4. I cannot possibly undergo it
5. I cannot undergo it

Thank you so much for your cooperation
